# Evaluation of in vivo antitumor effects of low‐frequency ultrasound‐mediated miRNA‐133a microbubble delivery in breast cancer

**DOI:** 10.1002/cam4.840

**Published:** 2016-07-27

**Authors:** Yanlei Ji, Zhen Han, Limei Shao, Yuehuan Zhao

**Affiliations:** ^1^ Department of Special DiagnosisShandong Cancer Hospital affiliated to Shandong UniversityShandong Academy of Medical SciencesJinanChina; ^2^ Department of Internal MedicineJinan Second People's HospitalJinanChina

**Keywords:** EGFR, miR‐133a, ultrasound‐targeted microbubble destruction

## Abstract

MicroRNAs (miRNAs), as a novel class of small noncoding RNAs, have been identified as important transcriptional and posttranscriptional inhibitors of gene expression. Ultrasound‐targeted microbubble destruction (UTMD) is a noninvasive method for microRNA delivery. We aimed to investigate the effect of UTMD of miR‐133a on breast cancer treatment. It has been reported that miRNA‐133a is involved in various cancers. miR‐133a was lowly expressed in breast cancer tissues and breast cancer cell lines MCF‐7 and MDA‐MB‐231. The miR‐133a expression was significantly upregulated under exogenous miRNA‐133a treatment in MCF‐7 and MDA‐MB‐231 cells analyzed by qRT‐PCR. Exogenous miR‐133a promoted the cell proliferation as determined by diphenyl tetrazolium bromide (MTT) assay and 5‐ethynyl‐2′‐deoxyuridine (EdU) staining. Epidermal growth factor receptor (EGFR) expression and Akt phosphorylation were significantly suppressed after miR‐133a transfection by western blot detection. We prepared the miR‐133a‐microbubble and injected it into breast cancer xenografts. The miR‐133a‐microbubble injection prolonged miR‐133a circulatory time by detecting the amount of miRNA‐133a in the plasma. No significant toxicity was observed on alanine aminotransferase (ALT) and aspartate aminotransferase (AST) levels at liver and albumin, blood urea nitrogen, or creatine kinase levels at kidney after miR‐133a‐microbubble injection. The tumor size of miR‐133a‐microbubble‐injected mice was smaller than that of the control group. Furthermore, the delivery efficiency of miR‐133a with low frequency was higher than that with common frequency. miR‐133a suppressed cell proliferation by suppressing the expression of EGFR and the phosphorylation of Akt. UTMD of miR‐133a inhibited the tumor growth and improved the survival rate in breast cancer mice. Our study provides new evidence that UTMD of miRNA is a promising platform for breast cancer therapy.

## Introduction

Breast cancer is one of the most frequently diagnosed cancers and a major cause of cancer‐related death for females worldwide 1. Local recurrence and distant metastasis resulted in poor prognosis 2. Abnormalities of various transcriptional and posttranscriptional regulators have been revealed to be associated with breast cancer. MicroRNAs (miRNAs) are endogenous small noncoding RNAs and are involved in posttranscriptional gene regulation and function as oncogenes and tumor suppressors 3. A number of miRNAs have been reported involving in cell proliferation 4 and cell viability 5 in breast cancer, such as miR‐205, miR‐2,1 and miR‐133.

Epidermal growth factor receptor (EGFR) is a receptor tyrosine kinase locating at the cell surface. EGFR is highly expressed in various cancers and involved in cell proliferation, migration, and viability during the process of various cancers, such as breast cancer 6. The activation of EGFR is closely associated with poor prognosis. More efforts have been directed at developing anticancer agents to interfere with EGFR activity. It has been shown that miR‐133a suppresses cell cycle and proliferation in tumorigenesis through targeting EGFR 7. In breast cancer cells, loss of miR‐133a resulted in aberrant cell invasion that is related with poor prognosis and low survival by targeting FSCN1 8. So, miR‐133a might be a potential therapeutic target for breast cancer.

It is important to use a noninvasive approach to deliver specific miRNA to target area safely and effectively. Ultrasound‐targeted microbubble destruction (UTMD) is a novel method of interest for gene delivery. UTMD is revealed to be effective about the delivery of small interfering RNA 9, plasmid DNA 10, or different drugs. However, the study on miRNA delivery by UTMD is limited 11. In the study, we investigated the efficiency of miRNA‐133a delivery by UTMD techniques and reveal whether or not the miR‐133a delivery to breast cancer can suppress tumor in vivo and in vitro.

In this subject, our results demonstrate that miR‐133a suppressed cell proliferation through directly regulating the expression of EGFR and the phosphorylation of Akt. miR‐133a‐microbubble prolonged miR‐133a circulatory time in vivo after intravenous injection . UTMD of miR‐133a with low frequency resulted in the decrease of tumor size and the increase of survival rate. This study provides evidence that UTMD is an effective noninvasive technique for miR‐133a delivery for breast cancer therapy.

## Materials and Methods

### Cell culture and transfection

MCF‐7 and MDA‐MB‐231 breast cancer cells were purchased from the Cell Bank of Shanghai Institute of Cell Biology, Chinese Academy of Sciences. Cells were maintained at 37°C and 5% CO2 in Dulbecco's modified Eagle's medium (DMEM) (Gibco RL, Grand Island, NY) supplemented with 10% fetal bovine serum (FBS), 100 U/mL penicillin, and 0.1 mg/mL streptomycin.

The miR‐133a mimic (miR‐133a), miR‐133a inhibitor (Inhibitor), and miR‐133a scramble (negative miRNA control) were designed and synthesized by RiboBio (Guangzhou, China). MCF‐7 and MDA‐MB‐231 cells were seeded in 6‐well plates at 50% confluence. miR‐133a, miR‐133a inhibitor, or miR‐133a scramble were diluted into 250 *μ*L Opti‐MEM medium at the concentration of 50 nmol/L, and 5 *μ*L Lipofectamine 2000 (Invitrogen, San Diego, CA, USA) was added into 250 *μ*L Opti‐MEM medium at room temperature. About 10 min, diluted miRNA and lipofectamine 2000 were mixed well, and then dispensed into plates. Fresh medium was added 6 h after transfection. The control cells were only treated with the same volume of lipofectamine.

### RNA isolation and quantitative real‐time PCR

The 10 clinical breast cancer tissues (breast cancer tissues) and the corresponding nearby noncancerous breast tissue (normal breast tissues) used in this study were obtained from patients. All patients whose breast cancer samples were obtained signed an informed consent approving the use of their tissues for research purposes after operation and the study was approved by the Research Ethics Committee at the Shandong Cancer Hospital affiliated to Shandong University. Breast tissues and cells were collected and total RNA was extracted with Trizol (Invitrogen) after 1 day, 2 day, 3 day, or 5 day transfection. The quality and quantity of RNA were determined by measuring the absorbance at 260 and 280 nm. Reverse transcription was performed using One Step Prime Script miRNA cDNA Synthesis kit (Takara). The expression of miR‐133a was analyzed by quantitative SYBR Green PCR kit (Qiagen, Germany). U6 small nuclear RNA was used for normalization. miR‐133a relative to U6 was determined using the 2^−ΔΔCT^ method.

### Western blot

Proteins were extracted from cells and the concentration was analyzed by Bradford assay. Equal amount of protein (50 *μ*g) were subjected to 7.5% (for EGFR) and 12.5% (for Akt phosphorylation) sodium dodecyl sulfate polyacrylamide gel electrophoresis (SDS‐PAGE) and blotted onto PVDF membranes (Millipore, Bedford, MA). The membrane was blocked in 2% nonfat milk in TBS (20 mmol/L Tris and 140 mmol/L NaCl; pH 7.5) at room temperature, rinsed three times with TBST (TBS + 0.2% Tween‐20), and then incubated overnight at 4°C with primary antibody. Afterward, the membrane was washed three times with TBST for 5 min each, and then probed with a secondary goat‐anti‐mouse IgG (Zhongshan Biotechnique, Beijing, China) (1:10,000 dilution in blocking buffer) for 2 h at room temperature. Subsequently, the membrane was washed three times with TBST for 10 min each and once in TBS for 10 min. The protein signal was detected using nitroblue tetrazolium (NBT) (Sigma Chemical Co, St. Louis, Mo, USA) and 5‐bromo‐4‐chloro‐3‐indolyl phosphate (BCIP) (Sigma Chemical Co, St. Louis, Mo, USA).

### MTT assay and EdU detection

Cell proliferation was measured by diphenyl tetrazolium bromide (MTT) assay and 5‐ethynyl‐2′‐deoxyuridine (EdU) detection with a MTT cell proliferation (Beyotime, China) and EdU assay kit (Invitrogen), respectively. Cells were seeded into 96‐well plates and transfected with miR‐133a, inhibitor or miR‐133a scramble, respectively. After 0, 24, 48, 72, and 120 h, cells were incubated with 10 *μ*L of MTT (5 mg/mL, Sigma) for another 4 h at 37°C, followed by removal of the culture medium and addition of 150 *μ*L dimethyl sulfoxide (DMSO). Absorbance values at a wavelength of 570 nm were recorded on a microplate reader.

After transfection, cells were exposed to 50 *μ*mol/L of EdU for 4 h at 37°C and fixed in 4% paraformaldehyde for 10 min at room temperature. After being washed with a phosphate‐buffered saline (PBS, 140 mmol/L NaCl, 2.7 mmol/L KCl, 10 mmol/L Na_2_HPO_4_, and 1.8 mM KH_2_PO_4_), and permeabilized with 0.2% TritonX‐100 in PBS at 37°C for 30 min at room temperature. After being washed with PBS twice for 5 min, cells were reacted with 100 *μ*L of 1 × Apollo reaction cocktail for 30 min. Then, the cells were stained with 100 *μ*L of Hoechst 33342 (5 *μ*g/mL) for 30 min and visualized under a fluorescent microscope.

### MicroRNA‐microbubble preparation

Cationic lipid microbubbles were prepared by sonicating an aqueous dispersion of 1 mg/mL polyethyleneglycol‐2000 stearate (PEG‐2000, Avanti, German), 2 mg/mL distearoylphosphatidylcholine (DSPC, Avanti, German), and 0.4 mg/mL 1,2‐distearoyl‐3‐trimethylammoniumpropane (DOTAP, Avanti, German) with perfluoropropane gas 12. The target miR‐133a was added into cationic lipid microbubbles, and the mixture was incubated on a flat rocker to facilitate miRNA‐microbubble interaction for 30 min.

### Tumor xenografting and ultrasound

Female athymic BALB/c nude mice (4–6 weeks old) were purchased from Shanghai Experimental Animal Centre, Chinese Academy of Science. Before MCF‐7 cells injection, estrogen pellets (IRA, Toledo, OH) with 60 days sustained release, containing 0.72 mg of estrogen were supplied to the animals subcutaneously. Three days later, breast tumor xenografts were obtained by subcutaneously injecting 4 × 10^6^ MCF‐7 cells suspended in 0.2 mL 0.9% NaCl into the nude mice. After about 3 weeks, palpable tumors were established and reached 190 mm^3^. The tumor volume (V) was calculated using calipers and calculated using the formula: m1^2^ × m2 × 0.5236, where m1 represents the shortest axis and m2 the longest axis. Then, the mice were divided into seven random groups for different treatments (*n* = 5, each group). G0, the control group was injected only with 0.9% NaCl; G1, the control group was injected only with 0.9% NaCl and ultrasounded with low frequency (1 MHz); G2, the group injected with scrambled‐microbubble (100 *μ*g) and ultrasounded with low frequency (1 MHz); G3, the group injected with miR‐133a‐microbubble (100 *μ*g) and ultrasounded with low frequency (1 MHz); G4, the group injected with miR‐133a‐microbubble (100 *μ*g) and ultrasounded with common frequency (10 MHz); G5, the group injected with miR‐133a microbubble (50 *μ*g) and ultrasounded with low frequency (1 MHz); G6, the group injected with miR‐133a microbubble (200 *μ*g) and ultrasounded with low frequency (1 MHz). The details are listed in Table 1. A single‐element transducer with a 1/2‐inch diameter aperture was used in the experiments. An acoustic pressure of 1 MPa at the focus with a 50% duty cycle and a sonication intensity of 0.9 w/cm^2^ was employed.

**Table 1:** The mice were divided into six groups and received different treatments

Group	Different treatments	Ultrasound
0	Control	Without
1	Control	1 MHz 20 min
2	Scramble‐miRNA‐MB (100 *μ*g)	1 MHz 20 min
3	miR‐133a‐MB (100 *μ*g)	1 MHz 20 min
4	miR‐133a‐MB (100 *μ*g)	10 MHz 20 min
5	miR‐133a‐MB (50 *μ*g)	1 MHz 20 min
6	miR‐133a‐MB (200 *μ*g)	1 MHz 20 min

### Statistical analysis

The results were expressed as mean ± SD. Means of different treatment groups were tested for statistical difference compared to the untreated control group with a Student's *t*‐test and considered significantly different at *P* < 0.05. Statistical analysis was performed with Prism5 (Graphpad Software, La Jolla, CA).

## Results

### miR‐133a suppressed cell proliferation through inhibiting EGFR expression and Akt phosphorylation

**Figure 1:**
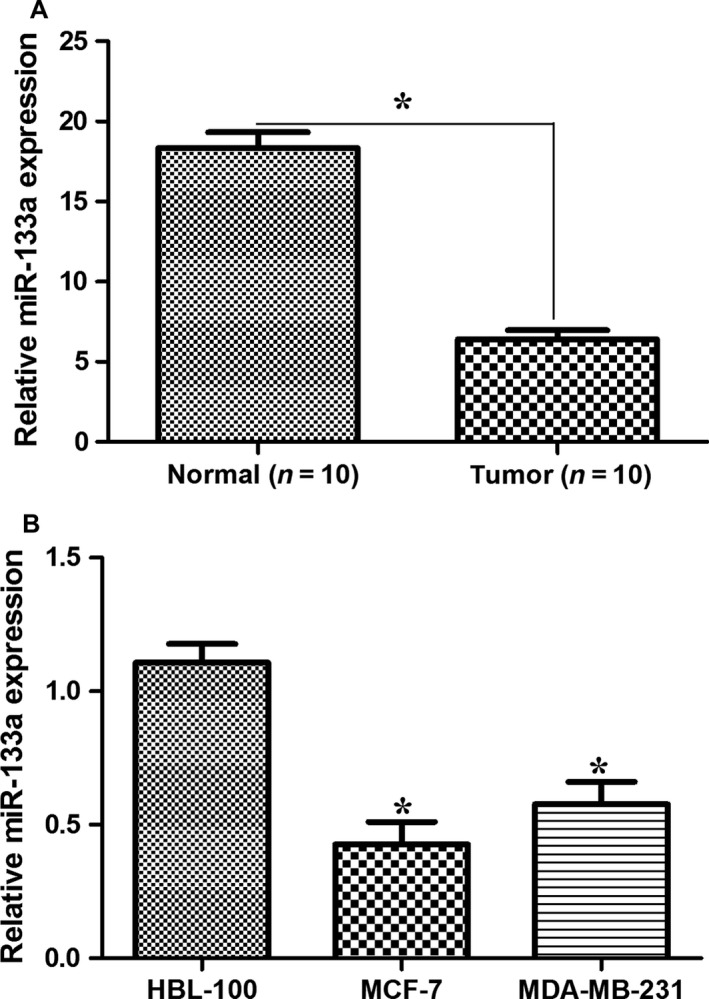
miR‐133a was suppressed in breast cancer tissues and cell lines. (A) Relative expression level of miR‐133a in breast cancer tissue samples and the normal tissue samples was analyzed by qRT‐PCR. We collected *N* = 10. (B) The levels of miR‐133a from different cell lines were detected by qRT‐PCR. HBL‐100, normal breast cell line.*P < 0.05, Student's *t*‐test.

We first analyzed the expression of miR‐133a in breast cancer tissues and cells by qRT‐PCR. Compared with normal breast tissues, miR‐133a was lowly expressed in breast cancer tissues (Fig. 1A). Similarly, the levels of miR‐133a in MCF‐7 cells and MDA‐MB‐231 cells were lower than that in normal breast cancer cells HBL‐100 (Fig. 1B). These results showed that miR‐133a was a suppressor in breast cancer.

**Figure 2:**
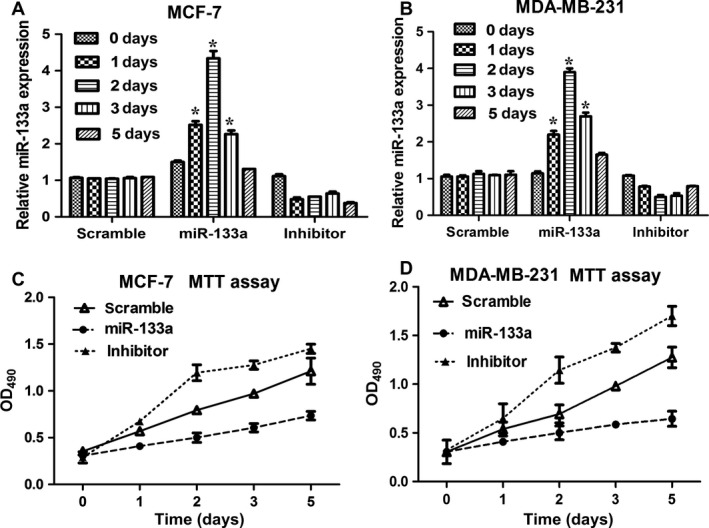
miR‐133a suppressed cell proliferation. The MCF‐7 and MDA‐MB‐231 cells were transfected with scramble‐miRNA, miR‐133a, and miR‐133a inhibitor, respectively. (A) The miR‐133a expression was detected after 0, 1, 2, 3, or 5 day in MCF‐7 (A) and MDA‐MB‐231 (B) cells by qRT‐PCR. *P < 0.05, Student's *t*‐test. miR‐133a suppressed cell proliferation in MCF‐7 (C) and MDA‐MB‐231 (D) cells analyzed with MTT assay.

**Figure 3:**
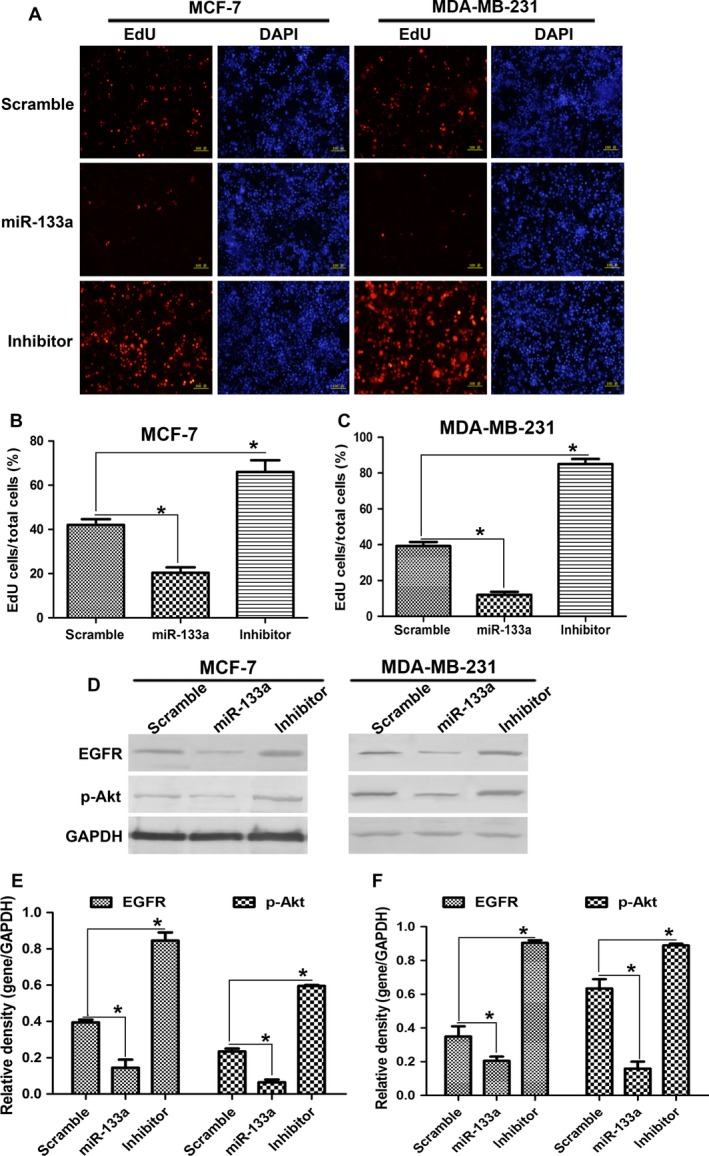
miR‐133a suppressed cell proliferation through inhibiting EGFR expression and Akt phosphorylation. The MCF‐7 and MDA‐MB‐231 cells were transfected with scramble‐miRNA, miR‐133a, and miR‐133a inhibitor, respectively. After 48 h, the cells were stained with EdU (A). The nuclei were stained with DAPI. Scale bar = 100 *μ*m. (B and C) were the statistical analysis of A. (D) miR‐133a transfection repressed the expression of EGFR and the phosphorylation of Akt analyzed by western blot. (E and F) were the statistical analysis of D. *P < 0.05. EGFR, epidermal growth factor receptor; MTT, diphenyl tetrazolium bromide; EdU, 5‐ethynyl‐2′‐deoxyuridine.

To examine the efficiency of miR‐133a transfection, we detected the level of miR‐133a from 0 to 5 day after miR‐133a incubation. In Figure 2A, miR‐133a level in MCF‐7 cells showed a peak expression at 48 h after miR‐133a transfection, and then declined, however, miR‐133a inhibitor obviously suppressed miR‐133a level. And scrambled miRNA transfection did not affect the miR‐133a expression. Similar results were found in MDA‐MB‐231 cells (Fig. 2B). Compared with scrambled miRNA transfection, exogenous miR‐133a transfection repressed cell proliferation, and miR‐133a inhibitor promoted cell proliferation when determined by MTT assay (Fig. 2C and D). EdU staining has been a sensitive and fast method to study cell proliferation 13. miR‐133a transfection obviously caused the decrease in cell numbers, compared with scrambled miRNA or miR‐133a inhibitor transfection (Fig. 3A, B and C). EGFR is reported to be a direct target of miR‐133a 7. Western blot results showed that miR‐133a suppressed the level of EGFR and the phosphorylation of Akt in MCF‐7 and MDA‐MB‐231 cells (Fig. 3D, E and F). These results revealed that miR‐133a repressed cell proliferation by negatively regulating EGFR expression and suppressing the phosphorylation of Akt.

### The miR‐133a‐microbubble injection prolonged miR‐133a circulatory time in vivo

**Figure 4:**
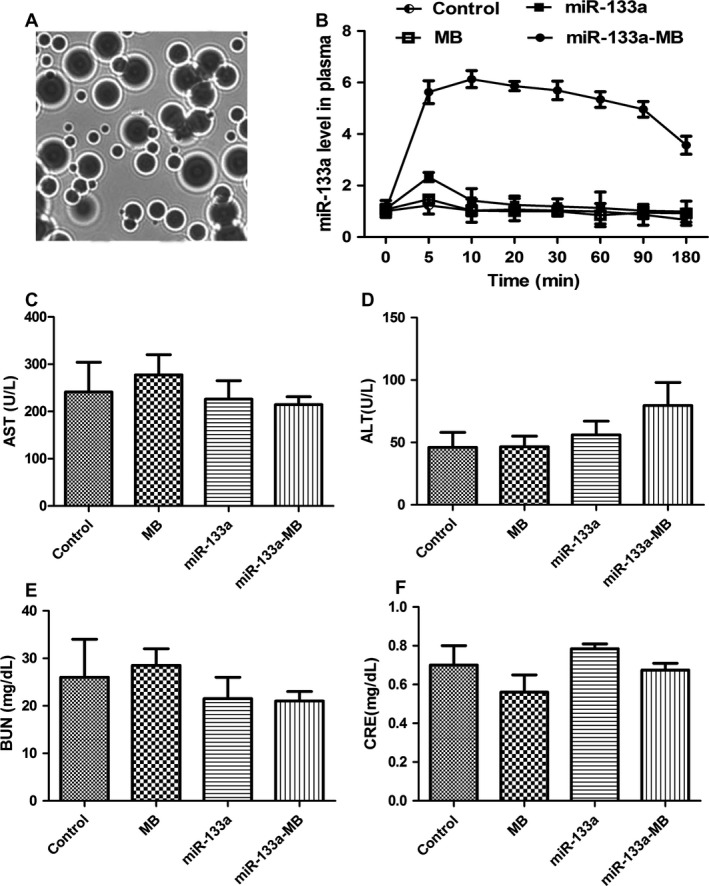
The miR‐133a in combination with microbubble prolonged miR‐133a circulatory time in vivo. (A) White light illumination of the microbubbles. (B) Time course of miR‐133a circulation in plasma after intravenous injection. MB, microbubble; miR‐133a‐MB, miR‐133a bound to microbubble. Data expressed as mean ± SD. Indices of acute toxicity in the kidney and liver tissues including AST (C), ALT (D), BUN (E), and CRE (F) were detected. ALT, alanine aminotransferase; AST, aspartate aminotransferase; BUN, blood urea nitrogen; CRE, creatine kinase.

Cationic microbubbles technique has been a useful method for miRNA delivery for therapeutic angiogenesis. We injected the athymic BALB/c nude mice with MCF‐7 cells to obtain breast tumor xenografts. We analyzed the expression of miR‐133a to investigate the role of miR‐133a in MCF‐7‐induced breast tumor mice. In supplemental Figure 1, the level of miR‐133a in nude mice tumor tissues was lower than that in normal nude mice, showing that miR‐133a played roles in breast tumor xenografts. Upon intravenous administration to mice, higher concentrations and longer circulatory time of miR‐133a were detected in the plasma after injection of miR‐133a‐microbubble (miR‐133a‐MB for short, Fig. 4A) compared with control, miR‐133a alone, or microbubble alone (MB, Fig. 4B). These results suggested that miR‐133a‐MB injection maintained the stabilization of miR‐133a and prolonged its circulation.

After different treatments, mice were killed and the blood samples were analyzed to evaluate liver damage and kidney toxicity. Compared with the control group, the levels of aspartate aminotransferase (AST) and alanine aminotransferase (ALT) were in the normal range of toxicity (Fig. 4C and D). And no significant differences between different treatments in blood urea nitrogen (BUN) or creatine kinase (CRE) levels were found (Fig. 4E and F). These findings showed that miRNA‐MB injection has no toxicity on the organism.

### The miR‐133a‐microbubbles delivered with low‐frequency ultrasound suppressed the tumor growth and improved the survival rate

**Figure 5:**
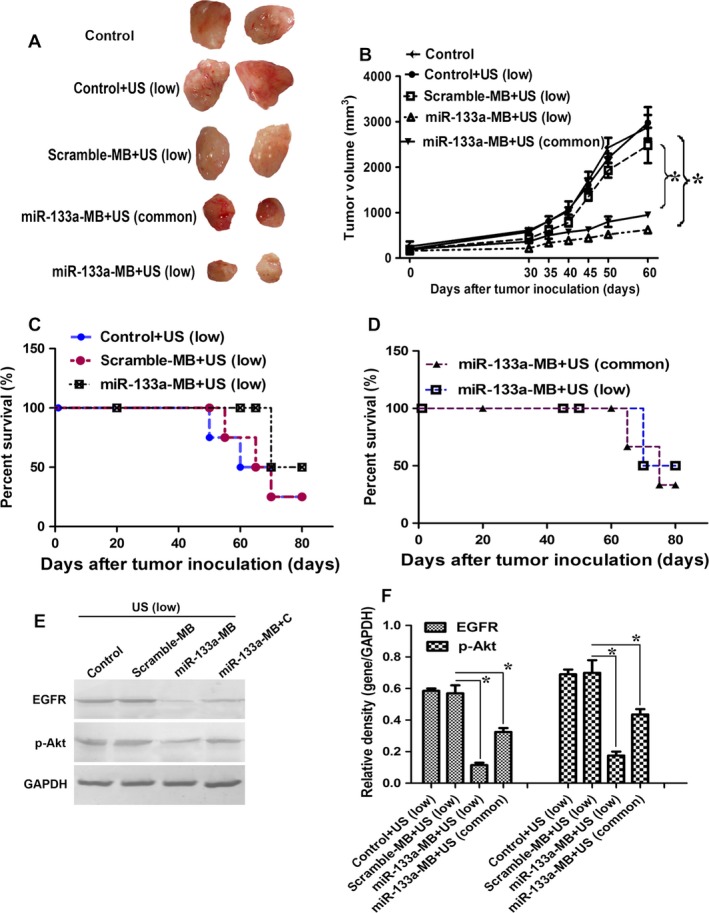
The miR‐133a‐microbubbles delivered by ultrasound with low frequency suppressed the tumor growth and improved the survival rate. (A) Images of isolated xenograft tumors after different MBs injection and different frequency ultrasound. **(**B) Tumor growth of human breast cancer xenografts treated with scramble‐MB or miR‐133a‐MB with a low frequency or common frequency ultrasound (each five mice per experimental group). *P < 0.05. (C) Survival rate of human breast cancer xenografts treated with scramble‐miRNA‐MB or miR‐133a‐MB delivery. (D) Survival rate of breast cancer xenografts after miR‐133a delivered with a low frequency or common frequency ultrasound. (E) The effect of scramble‐miRNA‐MB or miR‐133a delivery with different frequency ultrasound on EGFR expression and Akt phosphorylation analyzed by western blot. GAPDH was used as the interval control. (F) was the relative density analysis of E. *P < 0.05.

We achieved breast tumor xenografts by MCF‐7 cells injection. To determine the effect of miR‐133a on tumor size, we injected different MB with ultrasound and calculated the tumor volumes. There was no difference on tumor size between control mice (G0) and control mice with ultrasound (G1) (Fig. 5A and B. *P *>* *0.05). Scrambled‐miRNA‐MB had no effect on tumor growth. Compared with the scrambled‐miRNA‐MB‐injected mice (G2), the tumor size from miR‐133a‐MB‐injected mice (G3) was smaller (Fig. 5A and B). In order to reveal the effect of ultrasound frequency on the tumor suppression, we performed ultrasound on the miR‐133a‐MB‐injected mice with low frequency (G3, 1 MHz) or common frequency (G4, 10 MHz). Low frequency suppressed tumor growth more significantly (Figs. 5A and B).

In addition, the survival rate of each experimental group was evaluated by log‐rank test. There was no obvious significance between control+US and scrambled‐miRNA‐MB+US on survival rate. Compared with scrambled‐miRNA‐MB‐treated mice, the first death of miR133a‐MB‐injected mice was delayed for 7.5 day (67.5 day vs. 75 day, *P* = 0.037) (Fig. 5C). In order to investigate frequency of ultrasound on the survival rate, we scanned the miR‐133a‐MB‐injected mice with different frequency (1 MHz or 10 MHz). In Figure 5D, low frequency obviously improved the survival rate. These results mean that miR‐133a‐MB delivery with low‐frequency ultrasound effectively suppresses the tumor growth, and thereby increasing the survival rate.

We also analyzed the effect of different treatments on the expression of EGFR and the phosphorylation of Akt. Under the low‐frequency ultrasound, when the mice were injected with miR‐133a‐MB, the EGFR expression and the phosphorylation of Akt were significantly suppressed, compared with the group that received the same amount of scramble‐miRNA‐MB. Compared with groups that received miRNA‐133a‐MB with low‐frequency ultrasound or common frequency ultrasound, the low frequency obviously inhibited the expression of EGFR and the Akt phosphorylation in mice (Figs. 5E and F). These results show that low‐frequency ultrasound is more effective than common frequency for miR‐133a‐MB delivery.

### The outcome of miR‐133a delivery is in dosage‐dependent manner at some dosage range

**Figure 6:**
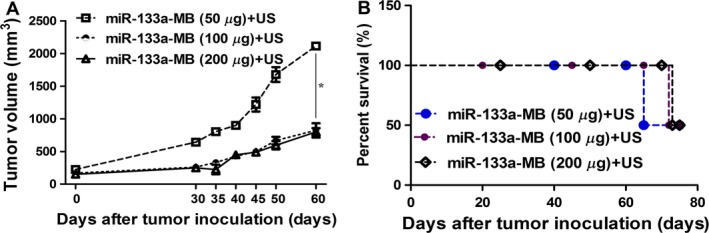
The tumor volume and survival rate of the xenograft model were affected by different dosages of miR‐133a‐microbubble in vivo. (A) Tumor growth of human breast cancer xenografts treated with miR‐133a at the concentration of 50, 100, or 200 *μ*g. (each five mice per experimental group). (B) The survival rate of human breast cancer xenografts treated with miR‐133a at the concentration of 50, 100, or 200 *μ*g. *P < 0.05. EGFR, epidermal growth factor receptor.

In order to further investigate the effect of miR‐133a‐MB dosage on breast cancer, we injected the mice with different dosages of miR‐133a‐MB (50, 100, or 200 *μ*g) and measured the tumor size and survival rate. Compared with 50 *μ*g miR‐133a‐MB injection, the first death of 100 *μ*g of miR‐133a‐MB injection was delayed for 3.5 day (73.5 day vs. 70 day, *P* = 0.045), which showed that the outcome of miR‐133a‐MB at 100 *μ*g injection is better than that of 50 *μ*g. However, there is no obvious significance between 100 *μ*g and 200 *μ*g. These findings revealed that the outcome of miR‐133a delivery is in dosage‐dependent manner at some dosage range (Fig. 6A and B).

**Figure 7:**
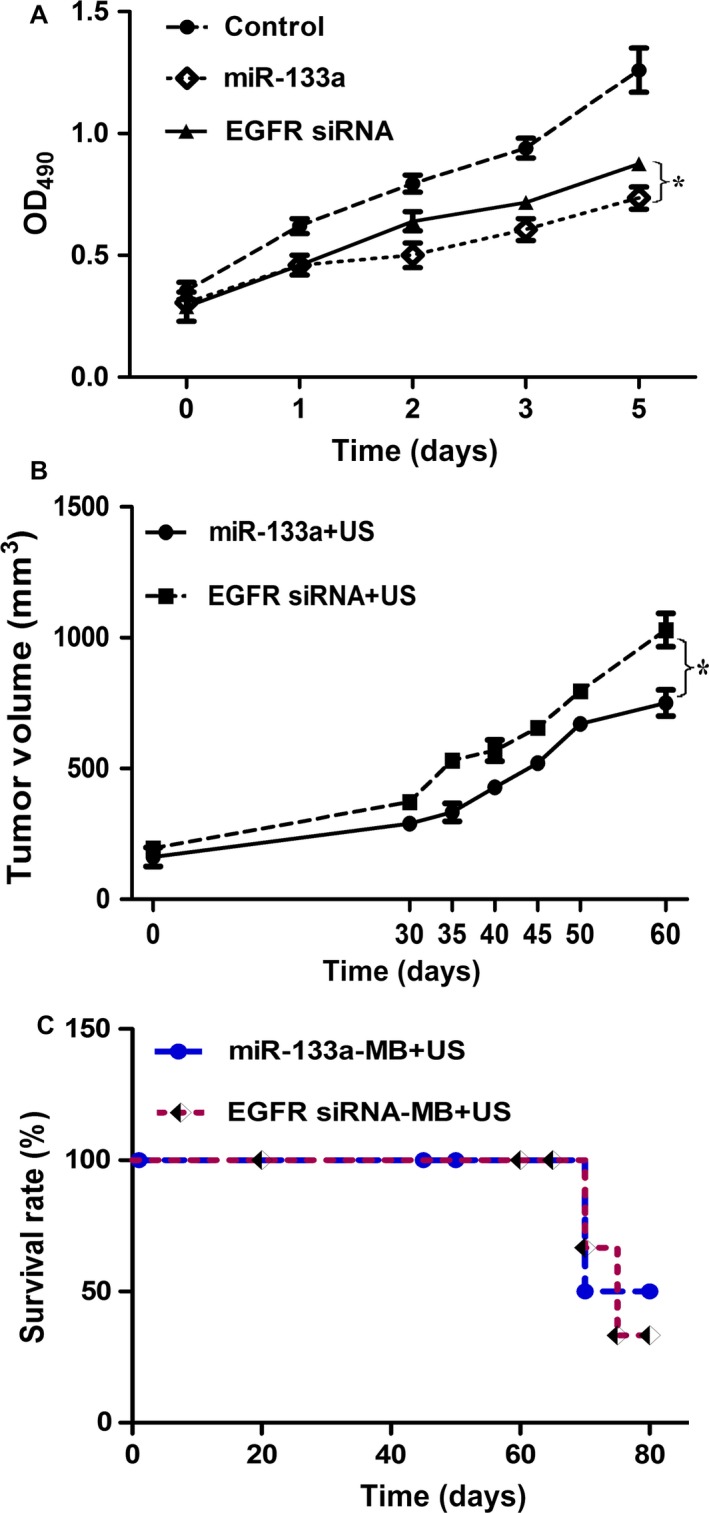
The diversity of EGFR siRNA or miR‐133a in vitro and in vivo. (A) The effect of EGFR siRNA or miR‐133a on cell proliferation. The MCF‐7 cells were transfected with the same amount of EGFR siRNA or miR‐133a, then the cell proliferation was detected with MTT assay. The effect of EGFR siRNA or miR‐133a on the tumor sizes (each five mice per experimental group) (B) and the survival rate (C). We delivered about 100 *μ*g EGFR siRNA‐MB or miR‐133a‐MB into mice with ultrasound, and examined the tumor sizes and the survival rate. *P < 0.05. EGFR, epidermal growth factor receptor; MTT, diphenyl tetrazolium bromide.

As we know, siRNAs and miRNAs are noncoding RNAs with important roles in gene regulation, and the therapeutic applications of siRNAs and miRNAs are popular. We compared the differences of two approaches in vitro and in vivo. EGFR is the direct target of miR‐133a. In vitro, we transfected EGFR siRNA or miR‐133a into MCF‐7 cells and investigated the effect on cell proliferation. In Figure 7A, both EGFR siRNA and miR‐133a suppressed cell proliferation, however, the inhibition of cell proliferation in miR‐133a‐transfected cells was more significant. In vivo, we injected EGFR siRNA‐MB or miR‐133a‐MB into mice and examined the tumor size and survival rate. The tumor size of miR‐133a‐MB injection was smaller than that of EGFR siRNA‐MB injection (Fig. 7B). The first death of miR‐133a‐MB‐treated mice was delayed 2 day than that of EGFR siRNA‐MB injection (Fig. 7C). These results showed that the outcome of miR‐133a is better than EGFR siRNA in tumor size and survival rate in this experiment, however, the difference of two approaches needs further investigation.

## Discussion

miRNAs negatively regulate target gene expression at the posttranscriptional level by binding to the 3′ untranslated region of mRNA 14. miRNAs play an important role in tumorigenic and metastatic progression. Deregulation of miRNAs has been linked to diverse pathological processes, including cancer 15. miR‐133a, which belongs to the miR‐133 family, was first identified as a muscle‐specific miRNA. Recently, a number of reports have shown that miR‐133a acts as a tumor suppressor in various cancers. In head and neck squamous cell cancer, miR‐133a regulated tumor cell migration and invasion by targeting caveolin‐1 16. miR‐133a induced apoptosis through direct regulating of GSTP1 in bladder cancer 17. In breast cancer, miR‐133a regulated cell cycle and proliferation by targeting EGFR through EGFR/Akt pathway 7. In our study, miR‐133a transfection suppressed cell proliferation, EGFR expression and Akt phosphorylation. These results were similar to the previous findings. So, miR‐133a might be a potential therapeutics for tumor treatment.

Blocking the function of specific miRNAs has been studied for several years, however, miRNA inhibitors have low organ specificity. Although many techniques have been explored, the optimal delivery method for miRNA remains to be well determined. Recently, some reports have shown that ultrasound technique combined with microbubble could enhance miRNA delivery to specific target tissues employing different frequencies 18. The most useful advantage of ultrasound as a therapeutic system is that ultrasound can focus on a specific area 19. In this research, we found that miR‐133a bound to microbubbles prolonged the lifetime of miR‐133a in the plasma. This finding showed that the microbubble could stabilize miR‐133a. The analysis of biochemical indexes revealed that the injection of miR‐133a‐MB had no toxicity on the mice. The tumor size was smaller, and the survival rate was prolonged in the miR‐133‐MB‐injected mice under ultrasound. That is to say, miRNA bound to microbubble delivery with ultrasound is feasible in tumor treatment. In this paper, we found the tumor size and survival rate were different under different frequency ultrasound. Therefore, the choice of frequency of ultrasound is important.

Both siRNAs and miRNAs aim to silence cancer‐related genes in order to suppress tumor cell proliferation and metastasis. In our study, we found that miR‐133a suppressed cell proliferation and tumor size, and increased the survival rate more significantly, compared with EGFR siRNA. These results refer to the different mechanisms of siRNA and miRNA on silencing the genes expression. One miRNA has various targets and it can potentially bind to whole groups of mRNA targets that are involved in the same processes to generate pronounced therapeutic effect 20. For example, miR‐133a can inhibit cell proliferation and invasiveness through directly suppressing the expressions of insulin‐like growth factor 1 receptor, TGF‐beta receptor type‐1, EGFR in non‐small cell lung cancer 21, and breast cancer 7. However, one siRNA is limited to target only one gene, so that the function is specific. Therefore, we should choose the proper methods to knockdown genes according to the objective.

## Conclusion

In this study, we found that miR‐133a transfection suppressed cell proliferation through repressing EGFR expression and Akt phosphorylation in vivo and in vitro. This finding is associated with the previous report. No significant toxicity was observed on ALT and AST levels at liver and ALB, BUN, or CRE levels at kidney by biochemistry indexes analysis. miR‐133a‐MB delivery using ultrasound led to tumor regression by knockdown of EGFR. The survival rate of experimental group using miR‐133a‐MB with ultrasound showed statistically significant increase compared to that of control group using scramble‐MB. The outcome of miR‐133a delivery at low frequency is more significant than that at common frequency.

## Conflict of Interest

None declared.

## Supporting information


**Figure S1.** miR‐133a was suppressed in breast tumor xenografts obtained by MCF‐7 cells injection into the athymic BALB/c nude mice. We isolated the breast tissues from nude mice or breast tumor nude mice. Total RNAs were extracted for qRT‐PCR. Normal mice, athymic BALB/c nude mice without treatment; Tumor mice, MCF‐7 cells induced breast tumor xenografts. **P < 0.01.

## References

[1] Jemal, A. , F. Bray , M. M. Center , J. Ferlay , E. Ward , and D. Forman . 2011 Global cancer statistics. CA Cancer J. Clin. 61:69–90. 21296855 10.3322/caac.20107

[2] Tan, X.‐F. , and F. Xia . 2014 Long‐term fatigue state in postoperative patients with breast cancer. Chin. J. Cancer Res. 26:12. 24653622 10.3978/j.issn.1000-9604.2014.01.12 PMC3937751

[3] Hata, A. , and R. Kashima . 2016 Dysregulation of microRNA biogenesis machinery in cancer. Crit. Rev. Biochem. Mol. Biol. 51:124–134. 10.3109/10409238.2015.1117054 PMC5226641 26628006

[4] Yu, Z. , C. Wang , M. Wang , Z. Li , M. C. Casimiro , M. Liu , et al. 2008 A cyclin D1/microRNA 17/20 regulatory feedback loop in control of breast cancer cell proliferation. J. Cell Biol. 182:509–517. 18695042 10.1083/jcb.200801079 PMC2500136

[5] Cui, W. , Y. Zhang , N. Hu , C. Shan , S. Zhang , W. Zhang , et al. 2010 miRNA‐520b and miR‐520e sensitize breast cancer cells to complement attack via directly targeting 3′ UTR of CD46. Cancer Biol. Ther. 10:232–241. 20574151 10.4161/cbt.10.3.12277

[6] Herbst, R. S. 2004 Review of epidermal growth factor receptor biology. Int. J. Radiat. Oncol. Biol. Phys. 59:S21–S26. 10.1016/j.ijrobp.2003.11.041 15142631

[7] Cui, W. , S. Zhang , C. Shan , L. Zhou , and Z. Zhou . 2013 microRNA‐133a regulates the cell cycle and proliferation of breast cancer cells by targeting epidermal growth factor receptor through the EGFR/Akt signaling pathway. FEBS J. 280:3962–3974. 23786162 10.1111/febs.12398

[8] Wu, Z‐S , C‐Q Wang , R. Xiang , X. Liu , S. Ye , X‐Q Yang , et al. 2012 Loss of miR‐133a expression associated with poor survival of breast cancer and restoration of miR‐133a expression inhibited breast cancer cell growth and invasion. BMC Cancer 12:51. 22292984 10.1186/1471-2407-12-51 PMC3297527

[9] Xu, Q. , T. Sun , H. Tian , C. Wang , and H. Zhou . 2013 Ultrasound‐mediated vascular endothelial growth factor C (VEGF‐C) gene microbubble transfection inhibits growth of MCF‐7 breast cancer cells. Oncol. Res. 20:297–301. 23879170 10.3727/096504013x13639794277680

[10] Taniyama, Y. , K. Tachibana , K. Hiraoka , T. Namba , K. Yamasaki , N. Hashiya , et al. 2002 Local delivery of plasmid DNA into rat carotid artery using ultrasound. Circulation 105:1233–1239. 11889019 10.1161/hc1002.105228

[11] Cao, W. J. , J. D. Rosenblat , N. C. Roth , M. A. Kuliszewski , P. N. Matkar , D. Rudenko , et al. 2015 Therapeutic angiogenesis by ultrasound‐mediated microRNA‐126‐3p delivery. Arterioscler. Thromb. Vasc. Biol. 35:2401–2411. 26381870 10.1161/ATVBAHA.115.306506

[12] Leong‐Poi, H. , M. A. Kuliszewski , M. Lekas , M. Sibbald , K. Teichert‐Kuliszewska , A. L. Klibanov , et al. 2007 Therapeutic arteriogenesis by ultrasound‐mediated VEGF165 plasmid gene delivery to chronically ischemic skeletal muscle. Circ. Res. 101:295–303. 17585071 10.1161/CIRCRESAHA.107.148676

[13] Salic, A. , and T. J. Mitchison . 2008 A chemical method for fast and sensitive detection of DNA synthesis in vivo. Proc. Natl Acad. Sci. 105:2415–2420. 18272492 10.1073/pnas.0712168105 PMC2268151

[14] Kim, V. N. 2005 MicroRNA biogenesis: coordinated cropping and dicing. Nat. Rev. Mol. Cell Biol. 6:376–385. 15852042 10.1038/nrm1644

[15] Lu, J. , G. Getz , E. A. Miska , E. Alvarez‐Saavedra , J. Lamb , D. Peck , et al. 2005 MicroRNA expression profiles classify human cancers. Nature 435:834–838. 15944708 10.1038/nature03702

[16] Nohata, N. , T. Hanazawa , N. Kikkawa , M. Mutallip , L. Fujimura , H. Yoshino , et al. 2011 Caveolin‐1 mediates tumor cell migration and invasion and its regulation by miR‐133a in head and neck squamous cell carcinoma. Int. J. Oncol. 38:209–217. 21109942

[17] Uchida, Y. , T. Chiyomaru , H. Enokida , K. Kawakami , S. Tatarano , K. Kawahara , et al. 2013 MiR‐133a induces apoptosis through direct regulation of GSTP1 in bladder cancer cell lines. Urol. Oncol. 31:115–123. 21396852 10.1016/j.urolonc.2010.09.017

[18] Zhang, L. , Y. Liu , G. Xiang , Q. Lv , G. Huang , Y. Yang , et al. 2011 Ultrasound‐triggered microbubble destruction in combination with cationic lipid microbubbles enhances gene delivery. J. Hua. Univ. Sci. Technol. Med. Sci. 31:39–45. 10.1007/s11596-011-0147-3 21336721

[19] Phillips, L. C. , A. L. Klibanov , B. R. Wamhoff , and J. A. Hossack . 2010 Targeted gene transfection from microbubbles into vascular smooth muscle cells using focused, ultrasound‐mediated delivery. Ultrasound Med. Biol. 36:1470–1480. 20800174 10.1016/j.ultrasmedbio.2010.06.010 PMC2930891

[20] Lam, J. K. , M. Y. Chow , Y. Zhang , and S. W. Leung . 2015 siRNA Versus miRNA as therapeutics for gene silencing. Mol. Ther. Nucleic Acids 4: e252. 26372022 10.1038/mtna.2015.23 PMC4877448

[21] Wang, L.‐K. , T.‐H. Hsiao , T.‐M. Hong , H.‐Y. Chen , S.‐H. Kao , W.‐L. Wang , et al. 2014 MicroRNA‐133a suppresses multiple oncogenic membrane receptors and cell invasion in non‐small cell lung carcinoma. PLoS ONE 9:e96765. 24816813 10.1371/journal.pone.0096765 PMC4016005

