# Supplementary figures and images for: Evaluation of in vivo antitumor effects of low‐frequency ultrasound‐mediated miRNA‐133a microbubble delivery in breast cancer
Source: Cancer Med. 2016 Jul 27;5(9):2534–43. doi: 10.1002/cam4.840 (PMC5055178; doi:10.1002/cam4.840)

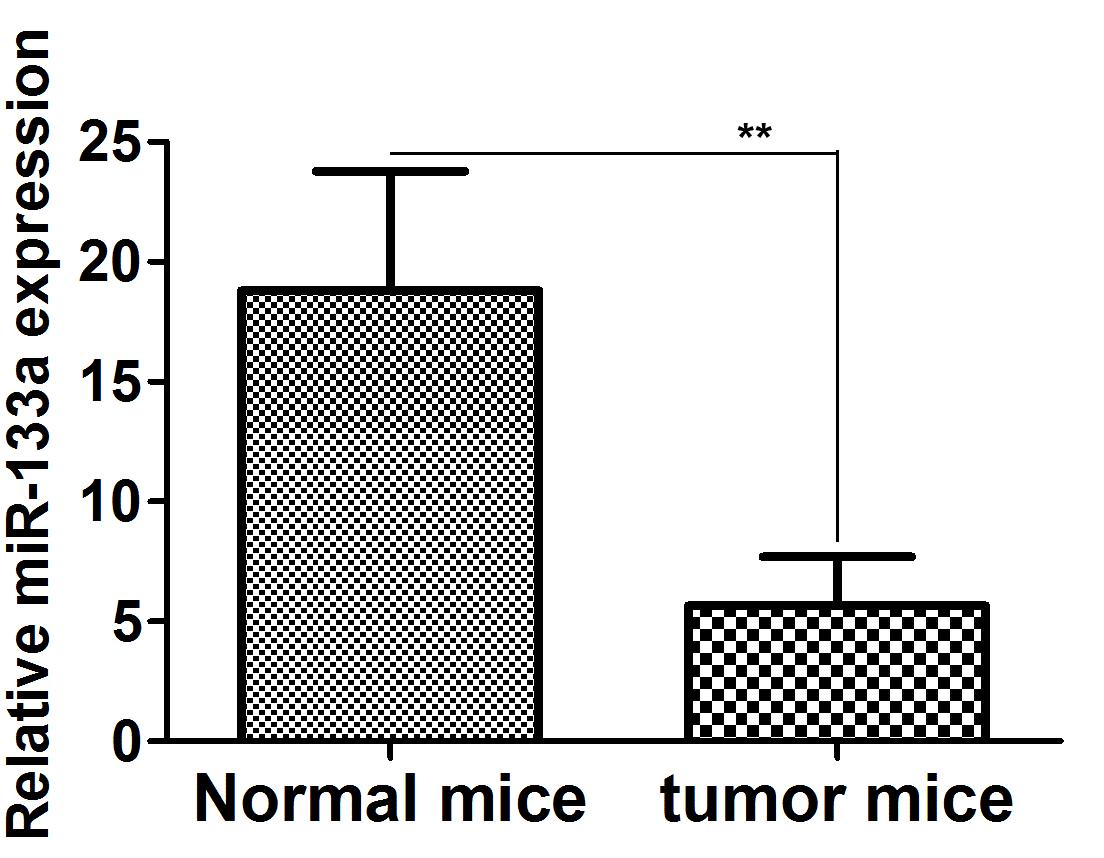

Supplement: Supplementary file 1 — Figure S1. miR‐133a was suppressed in breast tumor xenografts obtained by MCF‐7 cells injection into the athymic BALB/c nude mice. We isolated the breast tissues from nude mice or breast tumor nude mice. Total RNAs were extracted for qRT‐PCR. Normal mice, athymic BALB/c nude mice without treatment; Tumor mice, MCF‐7 cells induced breast tumor xenografts. **P < 0.01. [file CAM4-5-2534-s001.tif]
